# Effects of sucrose and lactose as partial replacement to corn in lactating dairy cow diets: a review

**DOI:** 10.1093/tas/txac044

**Published:** 2022-04-12

**Authors:** A D Ravelo, D Vyas, L F Ferraretto, A Faciola

**Affiliations:** 1 Department of Animal Sciences, University of Florida, Gainesville, FL 32611, USA; 2 Department of Animal and Dairy Sciences, University of Wisconsin-Madison, Madison, WI 53706, USA

**Keywords:** lactose, molasses, sucrose, whey

## Abstract

Carbohydrates are one of the three macronutrients that provides energy in diets and are classified by their structures. Starch is a nonstructural carbohydrate and polysaccharide made of glucose monomers used for storage in plant cells. When starch makes up greater than 30% of the DM in diets there can be adverse effects on NDF digestibility due to decreases in ruminal pH. Sugars are water soluble carbohydrates that consist of monosaccharide and disaccharide units. Sugars ferment faster than starch because microorganisms in the rumen can ferment carbohydrates at different rates depending on their structure; however, this has not been shown to have negative effects on the ruminal pH. Sources of sugars such as molasses (sucrose) or whey (lactose) can be included in the diet as a partial replacement for starch in dairy cow diets. The purpose of replacing starch with sugars in a diet would be to add differing sources of carbohydrates in the diet to allow for continual fermentation of carbohydrates by the microorganisms in the rumen. It has been seen in studies and previous literature that the partial replacement of starch with sugars has the potential to maintain the ruminal environment and milk yield and composition in dairy cows without reducing NDF digestibility. The objective of this review is to evaluate the effects of partially replacing starch with sugars in dairy diets and its implication on ruminal fermentation, nutrient utilization, milk production, and feeding replacement strategy.

## INTRODUCTION

Carbohydrates are one of the three macronutrients providing energy for continued bodily function and can constitute up to 70% of the lactating dairy cow diet (Erickson and Kalscheur, 2020). The importance of balancing types of carbohydrates in dairy cow diets is highly recognized as it can have effects on ruminal function and milk production. Carbohydrates are broadly classified into structural and nonstructural carbohydrates (NSC; NRC, 2001). Structural carbohydrates are fibrous components of the plant cell wall such as cellulose and hemicellulose which are fermented by microorganisms in the rumen (Hespell, 1988). Nonstructural carbohydrates are starch and sugars found in the inside of the plant cell, also known as the soluble portion of the cell, typically in the cytoplasm. These carbohydrates are more fermentable by microorganisms compared to structural carbohydrates (Ishler and Becker, 2021).

Starch is made of glucose monomers that can be fermented in the rumen by the microbial population. Starch fermentation in the rumen can provide precursors in the form of volatile fatty acids (VFA) for lactose, protein, and fat synthesis in the mammary gland (Ishler and Becker, 2021). Furthermore, VFA can provide energy to the dairy cow. Providing energy is essential to meet requirements for growth, maintenance, and production in the lactating dairy cow. It is recommended that diets contain 23% to 30% DM of starch (Sniffen, 2004). A potential downfall of feeding a high concentration of starch in the diet is that it could reduce dietary NDF concentration and decrease NDF digestibility through the associative effects of starch (Firkins et al., 2001; Ferraretto et al., 2013). Diets high in starch increase the production of VFA and lactate compared to the rate of clearance, driving down ruminal pH. Additionally, decreases fiber concentration decreases saliva production during eating allowing less salivary buffer production during that time (Jiang et al., 2017). This reduction in ruminal pH can decrease NDF digestibility by creating a less suitable environment for cellulolytic bacteria, leading to health impairments such as subacute ruminal acidosis and displaced abomasum (Krause and Oetzel, 2006).

The decline in NDF digestibility due to over feeding starch can be mitigated by the partial replacement of starch with sugars in the diet (Broderick et al., 2008). Sugars can be included in a lactating dairy diet at 4% to 8% of DM (Firkins et al., 2001). Even though sugars are more fermentable than starch, their inclusion in the diet, in recommended amounts, should not have detrimental effects on pH (Broderick et al., 2008; Chibisa et al., 2015) while helping maintain nutrient digestibility or in some cases even increasing NDF digestibility (Vallimont et al., 2004). An additional benefit of adding sugars in dairy cow diets would be the potential to include them through incorporation of sugar byproducts, such as molasses, and whey, into the diet. However, inclusion above recommended levels can cause a decrease in NDF digestibility, which is why there are reservations towards the inclusion of sugar or readily fermentable carbohydrates in dairy cow diets (Ishler and Becker, 2021). Achieving a combination of NSC in the diet is a challenge often undertaken due to the potential benefits to the animal from feeding a balance of starch and sugars. By feeding starch and sugars in combination there is the possibility of meeting energy requirements of the cow and maintaining productivity. The objective of this review is to evaluate the effects of replacing starch with sugars in dairy cow diets and its implication on ruminal fermentation, nutrient utilization, milk production, and dietary amounts.

## METHODS USED IN THE REVIEW

Google Scholar and PubMed searches were implemented to find articles relevant for describing the structure of different carbohydrates and their inclusion and use in dairy cow diets. More specifically journal articles that implemented the partial replacement of starch in the diet for sugars including sucrose, lactose, molasses, and whey were searched to summarize the findings in these experiments.

## CLASSIFYING CARBOHYDRATES

**Figure 1.**
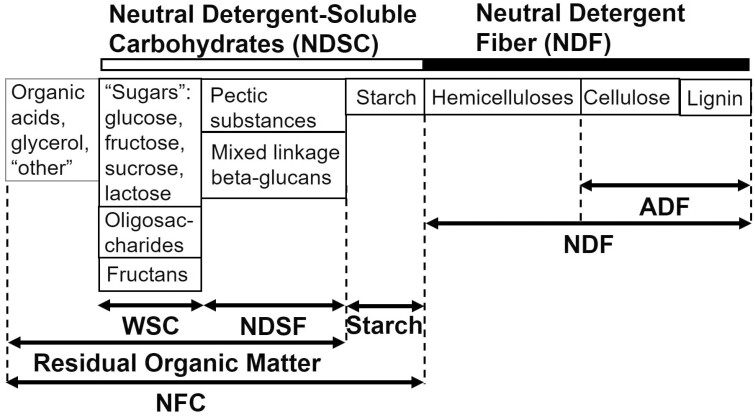
Carbohydrate fractions in feeds. ADF = acid detergent fiber, NDSF = neutral detergent-soluble fiber, WSC = water-soluble carbohydrates. Figure courtesy of M. B. Hall, U.S. Dairy Forage Research Center, USDA-ARS, Madison, WI.

Carbohydrates consist of monosaccharides, disaccharides, oligosaccharides, and polysaccharides. Monosaccharides include xylose, fructose, galactose, and glucose. Disaccharides are combinations of two monosaccharides. Disaccharides include sucrose (fructose and glucose) and lactose (glucose and galactose). Oligosaccharides are chains of 2 to 20 monomeric units of sugars (Roberfieod and Slavin, 2010), such as mannan oligosaccharides that can be included in the dairy cow diet (Guo et al., 2021). Polysaccharides are long chains of monomeric units of sugars. Common polysaccharides found in dairy cow diets would be cellulose, hemicellulose, and starch. Figure 1 illustrates the breakdown and classifications of carbohydrates found in feedstuffs. While cellulose and hemicellulose are considered components of the cell wall, starch and sugars are cell contents found inside the cell.

### Starch

Starch is made of glucose chains, and it is used for energy storage in plants (Pérez and Bertoft, 2010). It is classified under nonstructural carbohydrates and can make up to 70% of the DM found in corn grain (Ferraretto et al., 2013), a common feedstuff fed in cattle diets. Other feedstuffs rich in starch, include oats, barley, and sorghum grains. Starch consists of the straight chained polysaccharide amylose which has alpha 1,4-linkages and the branched chained amylopectin which has alpha 1,4 and 1,6 linkages (Pérez and Bertoft, 2010). Starch is fed as a common source of energy and its digestibility and utilization in the rumen can be affected by its particle size and degree of gelatinization (Hall and Mertens, 2017). Starch degradability in the rumen is important to consider because the more available the starch is, the more energy the microorganisms will be able to obtain from the feed.

When feeding cows, grains are usually processed to aid in their digestibility. Processing will make starch more available to the microorganisms and thus more available to the animal (Theurer, 1986). Decreasing the particle size of grains through mechanical means will increase microbial access to starch reserves (Rémond et al., 2004). However, dry rolling or grinding alone have less of an effect on starch digestibility when compared to pressure and moisture treatments. Processing of grain with the addition of heat and moisture can change the crystalline structure of starch to its gelatinized form making starch more susceptible to microbial fermentation (Rahini et al., 2020). Steam-flaking improves the digestion of corn, barley, and sorghum (Safaei and Yang, 2017). However, over processing increases the proportion of fine particles in the rumen and can cause digestive disorders such as acidosis and bloat (Koenig et al., 2013). Impacts of fine particles are also dependent on how much is fed and other dietary components. Hence processing focuses on maximizing starch digestion to allow for diet formulations that avoid digestive disturbances.

Health issues may arise with excess inclusion of starch in the diet from associated effects of decreased NDF, which is the fiber component of the cell and includes hemicellulose and cellulose components that are structural carbohydrates. More concentrate and less fiber in the diet yields an increase in VFA concentration which when paired with no change in the clearance of VFA from the rumen can decrease the ruminal pH (Penner et al., 2009). Milk fat percentage is sensitive to pH changes (Koch and Lascano, 2018), if milk fat is decreased that could be indicative that a drop in ruminal pH associated with acidosis has occurred. Milk fat can also be decreased through fatty acids such as linoleic acid coming from sources like corn oil. Thus, there needs to be a balance between dietary NDF and meeting energy requirements of the lactating dairy cow to optimize the ruminal environment. One way to mitigate this is by replacing starch with more readily fermentable carbohydrates such as soluble sugars due to sugars having the ability to provide greater fermentable energy for microbial protein production (Oba, 2011). The replacement of starch with sugars could help prevent negative effects of greater concentrations of starch in the diet and can potentially increase NDF digestibility and increase DMI.

### Soluble carbohydrates

Sugars are soluble carbohydrates as they are soluble in the cell’s aqueous environment (Maness, 2010). They are the part of NSC that does not include starch. Unlike starch, processing methods are not directed at soluble carbohydrates since they already exist in a form that is available to microorganisms and enzymes. They can typically be soluble in water (McDonald and Henderson, 1964) or some in ethanol. There are different approaches to feeding soluble carbohydrates to dairy cows. They can be fed directly as sugar; for example, sucrose and lactose are two disaccharides that have been fed directly in diets, or they can be incorporated through byproducts. Molasses and whey are examples of such byproducts that can be added to diets to increase sugar concentration.

Water-soluble carbohydrates (WSC) provide a source of readily available energy to microorganisms in the rumen. Unlike structural carbohydrates which need more time to be fermented by bacteria, WSC are fermented at a faster rate (Lee et al., 2003). Starch is fermented at a slower rate compared with WSC (Oba et al., 2015), but at a faster rate compared with structural carbohydrates (Moran, 2005). These rate differences are due to the types of bonds present in the sugars which impact ruminal degradability. The rate at which carbohydrates are consumed and fermented as well as the amount are important because it provides energy for continual microbial growth. With energy microorganisms can capture nitrogen from feeds and convert it into microbial protein that ruminants can later absorb and metabolize.

In an in vitro study by Lee et al. (2003) using a semi-continuous culture system, WSC concentration was increased by 25%, 50%, and 75% compared with a basal grass diet, and it was observed that increasing WSC decreased pH and ammonia (NH_3_–N). The authors also observed that increasing WSC concentration by 50%, increased microbial N concentration (158 vs. 144.6 mg/d) and the efficiency of microbial protein synthesis (12.7 vs. 9.9 g of N/kg of OM apparently digested) compared with the basal grass diet. The changes observed indicate a potential for improvement in microbial protein growth and efficiency with increasing WSC concentration.

Supplemental dietary sugar has been shown to increase butyrate concentrations in the rumen (Heldt et al., 1999; DeFrain et al., 2006; Martel et al., 2011). Butyrate has been shown to be beneficial for the growth and development of the ruminal epithelium and papillae (Rémond et al, 1995) which could improve short chain fatty acid absorption and pH regulation. Additionally, butyrate increases the substrates available for de novo milk fat synthesis. Hence, when fed with recommended concentrations of NDF (minimum of 25% of dietary DM with 19% of DM coming from forage, (NRC, 2001)), sugars can be incorporated as a replacement for starch to help prevent a decrease in NDF digestibility while achieving energy requirements.

## REPLACING STARCH WITH SUGARS

The interest in incorporating sugars into dairy cow diets as a replacement for starch stems from the benefit in meeting energy requirements while maintaining ruminal fermentation and production. Since corn grain is typically the main source of starch, when replacing starch with sugars, corn, or corn starch will typically be the feedstuff replaced. Replacing corn can help improve farm profitability and increase supply for human consumption. Incorporating sugars in the diet as a replacement for starch has the potential to increase DMI, maintain ruminal pH, and improve microbial protein synthesis, and milk components (Oba, 2011). Sugars that can be incorporated into the diet as a replacement for starch will typically be sucrose or lactose.

### Sucrose

Sucrose is a fructose and glucose bonded by an α-1,4 glycosidic bond (Campbell et al., 2005). Sucrose can be included in dairy cow diets as a source of readily fermentable energy. Vallimont et al. (2004) used a dual-flow continuous culture system to study the effect of partial replacement of starch with increasing amounts of sucrose (0%, 2.5%, 5%, and 7.5% of DM of the diet) on ruminal fermentation. The authors observed no changes in ruminal pH between the diets but a quadratic effect on NDF digestibility was noted, with the diet that had the greatest inclusion of sucrose (7.5% DM) having greater NDF digestibility. There was a linear increase in butyrate concentration in the fermenters with increasing sugar concentrations, but no differences were observed on N metabolism. The addition of sucrose to the diet did not affect microbial fermentation as long as there was sufficient rumen degraded protein, as suggested by the increase in NDF digestibility and evaluation of the diets fed using the Cornell Net Carbohydrate Protein System (V4.026). Results showed that sucrose can be fed without negatively impacting the ruminal environment. However, when sucrose was added at the greatest level of inclusion, then the microbial populations had less effective utilization of NH_3_–N. Hence, sucrose can be included in the diet, to a certain extent, without compromising bacterial fermentation in the rumen.

Diets partially replacing starch for sucrose using the same percentages as the ones tested by Vallimont et al. (2004) were tested by Broderick et al. (2008), who fed two sets of 24 lactating Holstein cows (summarized in Table 1). There were no changes in pH observed; however, there was a quadratic effect on NDF digestibility in which digestion was greatest at 5% sucrose inclusion. There was no change in total VFA or butyrate concentrations. The authors reported that as sucrose replaced starch, DMI and milk fat yield increased linearly. The DMI with 5.0% inclusion of sucrose increased by 1.5 kg/d compared with the control. With 2.5% inclusion of sucrose, milk production increase by 1.8 kg/d when compared with the control. Authors also observed a linear decrease in NH_3_–N concentration from 13.9 mg/dL in the diet with no sugar inclusion to 11.5 mg/dL in the diet with 7.5% of sugar inclusion. This could be indicative of more N being utilized for microbial protein synthesis. There was also a decrease in urinary excretion of urea N and total N. Overall, replacing starch with sugars or WSC could potentially lead to improvements in production and utilization of nitrogen.

**Table 1.** Summary of four experiments that tested the effects of replacing starch with sugars and measured ruminal parameters and production performance

Study and diet	Inclusion rate of sugar, % of the diet DM	Ruminal pH	Total VFA, mM	Buty^1^	Ammonia N, mg/dL	DMI, kg/d	NDF apparentdigestibility, %	Milk, kg/d	Milk fat, %
Broderick et al. (2008)									
7.5% starch/0% sucrose	0% sucrose	6.19	114.0	15.3	13.9^a^	24.5^b^	49.8	38.8	3.81^b^
5.0% starch/2.5% sucrose	2.5% sucrose	6.16	117.3	15.4	13.7^a^	25.4^ab^	52.6	40.6	3.80^b^
2.5% starch/5.0% sucrose	5.0% sucrose	6.18	114.1	15.5	12.4^ab^	26.0^a^	65.1	39.4	4.08^ab^
0% starch/7.5% sucrose	7.5% sucrose	6.21	111.3	15.4	11.5^b^	26.0^a^	54.2	39.3	4.16^a^
SE		0.11	5.0	0.7	0.9	0.5	3.9	0.9	0.12
Chibisa et al. (2015)									
Low sugar/barley	0% DWP^2^	6.03	111.0	10.4	13.1	28.2	43.3	41.0	3.51
Low sugar/corn	0% DWP^2^	6.09	110.0	11.2	14.9	29.7	49.6	40.2	3.54
High sugar/barley	6% DWP^2^	6.05	112.0	12.1	11.5	29.7	45.3	40.5	3.49
High sugar/corn	6% DWP^2^	6.17	105.0	12.4	10.8	29.8	45.4	40.9	3.41
SEM		0.14	3.6	0.54^3^	1.06^3^	0.96	1.87	2.36	0.13
Baurhoo and Mustafa (2014)									
0% molasses	0% DMol^4^	5.92	124.5	13.07	19.02	23.12	44.09	33.88	4.15
3% molasses	2.8% DMol^4^	5.97	121.08	12.91	21.64	23.75	44.16	32.77	4.12
6% molasses	5.6% DMol^4^	5.97	119.47	13.35	20.34	22.93	43.21	32.27	4.28
SEM		0.03	4.7	0.92	1.26	0.68	4.32	1.44^5^	0.14
(DeFrain et al., 2004)^6^									
CON	0% lactose	6.69	94.5	13.9	5.55	21.7	—	25.7	3.37
WHEY	9.4% liquid whey	6.68	98.4	16.1	4.11	22.6	—	24.9	3.38
LOLAC	7.1% lactose	6.78	97.6	16.3	4.57	22.3	—	25.8	3.48
HILAC	14.2% lactose	6.68	104.3	18.0	5.02	23.3	—	25.5	3.35
SEM		0.074	4.4	0.38^7^	0.65	1.16	—	2.4	0.086

Means with different superscripts within column and study differ significantly (*P* < 0.05).

Buty = butyrate (Broderick et al., 2008 reported in m*M*; Baurhoo and Mustafa, 2014, Chibisa et al., 2015, and DeFrain et al., 2004) reported as a percentage of total VFA).

DWP = dried whey permeate.

Significance noted in the sugar contrast (*P* < 0.05).

DMol = dried molasses.

Significance (*P* < 0.05) with no a, b subscripts provided.

No NDF digestibility data presented.

Contrasts used, significance (*P* < 0.05) noted between the control and diets that included sugar.

In another study by Penner and Oba (2009), 4.8% of DM of cracked corn grain was replaced with 4.7% of DM of sucrose and fed to 52 lactating Holstein cows. It was observed that the inclusion of sucrose in the diet tended to increase the mean ruminal pH from 6.06 to 6.21, and there were no treatment differences in NDF digestibility or VFA concentrations. Feeding the high sugar diet the first 4 weeks of lactation also increased DMI by 1.1 kg/d when compared with the low sugar diet. Milk fat yield tended to increase in cows fed the high sugar diet. It was concluded that when sugar was included in the diet there was an increase in DMI and milk fat yield in postpartum cows. There was also the potential for sucrose inclusion to reduce the severity of ruminal acidosis. Thus, it was summarized that sucrose could improve productivity when replacing cracked corn. From the studies summarized above, it can be concluded that to an extent the inclusion of sucrose as a replacement for starch is a viable option to improve nutrient digestibility and production, without detrimental effects on pH.

### Lactose

Similar to sucrose, lactose can be included in dairy cow diets. Lactose is a galactose and glucose connected by a β 1,4-glycosidic bond (Campbell et al., 2005). Lactose comes from milk; it is synthesized in the mammary gland of cows using two glucose molecules. One of the molecules is used as is while the other is converted into a galactose molecule and later bonded together and secreted in the milk. Compared with sucrose, there is a smaller decrease in ruminal pH caused by lactose inclusion in ruminal fluid (Weisbjerg et al., 1998). This could be explained by a slower hydrolysis of lactose compared with sucrose due to slower fermentation of galactose in comparison to glucose and fructose.

DeFrain et al. (2004) evaluated the effects of pure lactose and liquid whey inclusion using eight multiparous Holstein cows and four multiparous Brown Swiss cows (summarized in Table 1). The study had a control diet that included 12.1% DM of corn grain and 7.1 % DM of corn starch. To create a low lactose diet, the authors removed the 7.1% of DM coming from corn starch and replaced it with 7.1% DM of pure lactose. To create the high lactose diet, the authors removed corn starch and decreased corn grain inclusion to 2.4% of DM allowing an inclusion rate for pure lactose of 14.2%, double the concentration of the low lactate diet. No differences in pH or NH_3_–N were observed, but there was an increase in butyrate proportion and a decrease in the proportion of acetate and branched chained fatty acids in the sugar diets. With the increase of ruminal butyrate, there was also an increase in the plasma concentration of β-hydroxy butyric acid (BHBA); however, this increase was not enough to place cows at risk for developing ketosis. Additionally, the DMI of cows tended to increase linearly, intake was increased by 1.6 kg/d when comparing the control to the high lactose diet. However, it was noted that the greatest effect when feeding lactose is its apparent ability to change ruminal fermentation not necessarily through an increase on DMI; hence, there have been other reports that do not note an increase in DMI (DeFrain et al., 2006).

In the study by DeFrain et al. (2006), 24 multiparous transition Holstein cows were fed lactose to assess its effects on metabolic status. Cows were fed either a corn-based control diet or a diet with 15.7% of DM coming from lactose 21 days pre- and postpartum. There was an increase in rumen butyrate (9.2 vs. 11.3 molar proportion) and serum BHBA (7.65 mg/dL vs. 10.06 mg/dL) in cows fed the lactose compared to the control diet. This increase in ruminal butyrate concentration could potentially increase the amount of substrate available for milk fat synthesis and increase the fat percentage in milk composition; however this was not observed. There was suggestion of improved absorption capacity in the rumen from feeding lactose attributed to increased butyrate production since it can stimulate papillae development and growth increasing absorptive capacity. Additionally, it was concluded that with decreases in ruminal NH_3_–N and MUN, it is possible that there is an opportunity for greater microbial protein synthesis from lactose. Furthermore, the addition of lactose to the prepartum diets can have positive effects on metabolic status of the transition cow. As seen in the studies discussed here, the inclusion of lactose in the diet has the potential to increase butyrate concentration and maintain ruminal pH, nutrient digestibility, and production.

### Byproducts

Benefits from feeding byproducts can be economical if the byproduct is cost effective and increases economical returns through increased production. Molasses is a feedstuff that is a byproduct of sugar production that can be fed to ruminants as is or dried. Molasses can add palatability to the diet to decrease sorting behavior and increasing DMI and milk yield as a result (DeVries and Gill, 2012). Whey is another byproduct that is produced during the cheese making process. It can be fed to cattle as a liquid, dried, delactosed, or as a permeate (Schingoethe, 1980). There may be less consistency of available product in liquid whey and permeate compared to commercial dried and delactosed whey, therefore it is important to monitor the chemical composition. Treatment and processing of these byproducts could help improve self-life. Ravelo et al. (2021) evaluated the effects of partially replacing starch with molasses, condensed whey permeate, or a treated condensed whey permeate (treated with sodium hydroxide) in vitro and reported that the treatment of condensed whey had no changes in ruminal microbial fermentation and helped maintain pH.

Broderick and Radloff (2004) fed molasses as a supplement to lactating cows fed alfalfa and corn silage-based diets. The experiment had two trials with 48 Holstein cows blocked into 12 groups assigned to diets containing four levels of dried (trial 1) or liquid (trial 2) molasses. In the dried molasses trial, DMI increased 1 kg/d with 8% molasses inclusion and milk yield increased 0.9 kg/d compared with control. In the liquid molasses trial, there was a 2.7 kg/d increase in DMI with 3% liquid molasses inclusion compared to no liquid molasses, and milk yield was increased by 1.9 kg/d. Apparent digestibility of DM increased 4.2% with 12% dried molasses compared with the control. There was a tendency for decreased ruminal NH_3_–N when dried molasses was fed compared with no inclusion. A decrease in ruminal NH_3_–N can lead to less nitrogen being available to the fiber digesting bacteria; however, NDF digestibility was not affect in this trial and even increased with the inclusion of dried molasses. The authors concluded that overfeeding sugars appeared to reduce animal performance and optimum total dietary sugar for milk component yield and production was 5% total sugar by adding 2.4% sugar from liquid molasses to a diet containing 2.6% sugar.

In the study by Martel et al. (2011), two trials were conducted to assess the effects of partial replacement of starch with molasses on de novo fatty acid synthesis and ruminal traits. In the first trial, dietary molasses replaced corn grain at 0%, 2.5%, and 5% of DM in diets fed to 12 s-lactation Holstein cows. In the second trial, seven ruminally cannulated Holstein cows were used in a split plot cross over design with diets containing 0% or 5% molasses. In trial 1, milk fat concentration increased by 0.40% when molasses was fed from 0% to 5% inclusion in the diet. For trial 2, milk fat concentration increased 0.22% when molasses was fed from 0% to 5% inclusion in the diet. There was not an increase in milk yield observed in either trial. Dietary inclusion of molasses increased ruminal pH (5.73 vs. 5.87), decreased total VFA concentration (140.8 vs. 132.7), and promoted maintenance of normal ruminal fatty acid biohydrogenation through decreased acid load and abundance of protozoa, which promotes mammary de novo synthesis of fatty acid. Overall, the inclusion of sucrose or molasses did not compromise microbial fermentation and maintained production in lactating dairy cows.

The study by Baurhoo and Mustafa (2014) used 12 Holstein cows to evaluate the effects of partially replacing high moisture corn with 0%, 3%, or 6% of DM as supplemental dried molasses in alfalfa silage based diets (summarized in Table 1). A 1.61 kg/d decrease in milk yield was observed with 6% dried molasses compared with control. When feeding sugars, the increase in milk yield has been associated with an increase in DMI. In this study, there was a tendency for increased DMI with the 3% dried molasses supplementation compared to the 6% supplementation. The lower DMI observed with the 6% supplementation with dried molasses can help explain the decrease in milk yield observed. With 3% dried molasses supplementation, there was also a decrease in milk yield with a 1.11 kg/d reduction compared to no supplementation. Overall, milk yield decreased with both levels of inclusion.

Similar to molasses, whey can be fed to cattle as well. In a study by Chibisa et al. (2015), starch from corn or barley was replaced with dried whey permeate (DWP) (summarized in Table 1). The study used eight lactating cows in a 4 × 4 replicated Latin square design in which the cows were fed starch from either barley or corn with DWP supplemented at 0% or 6% of DM. Whey permeate is produced by the removal of protein and other solids from whey by ultrafiltration. Therefore, when dried, whey permeate consists mostly of lactose. Results from this study indicated that partial replacement of DWP for starch did not affect ruminal pH or plasma BHBA concentration. There was a reduction in NH_3_–N concentration in the high sugar diet which could indicate an improvement to nitrogen utilization; however, there was no increase in milk yield. Butyrate concentration was also increased in the high sugar diets.

In the study by De Seram et al. (2019), the effects of replacing barley starch with lactose were tested in a 4 × 4 Latin Square with eight cows, four of which were cannulated. Dried whey permeate was incrementally added in the diets to create diets with 0%, 3.8%, 7.6%, 11.5% of DM as DWP replacing barley grain. There were no effects of sugar inclusion on DMI or milk yield. Ruminal pH was not affected by diet. Butyrate concentration in the rumen increased with increasing DWP inclusion (12.8 mmol/L in control and 14.9 mmol/L in 11.5% DWP); however, there was no difference in plasma BHBA. There was decreased NH_3_ concentration in the rumen with increased sugar inclusion, 12.1 mg/dL in the control diet and 9.4 mg/dL in the diet with 11.5% DWP. There were improvements to N utilization until inclusion the level of 7.6% DWP, with the highest replacement they observed negative effects in factors relating to N utilization. Thus, it was concluded increasing levels of DWP replacement did not have a positive effect on milk protein or N balance.

Another recent study by Bernard et al. (2020) evaluated the effects of whey permeate inclusion in early- and mid-lactation dairy cows diets. Two trials were conducted with 48 Holstein cows. The first trial was 10 weeks long during mid-lactation, while the second trial was 12 weeks long during early-lactation. Both trials fed a control diet with no whey permeate and two other diets that included whey permeate at 3.1% and 6.1% DM of the diet as a replacement for finely ground corn. The cows in trial 1 tended to have increased DMI when fed the whey permeate, but no differences were observed in the second trial. No differences were observed among treatments in either trial. Overall, it was concluded that finely ground corn could be replaced by whey permeate with no adverse effects on production performance. However, when diets contained 6% whey permeate (DM basis), there was a tendency for reduced milk protein and lactose yield. Additionally, due to increases in DMI in the first trial with no difference in milk production the mid-lactation cows in this trial had reduced feed efficiency, but early lactation cows were not affected. Differing results between experiments can be due to consistency variations in products since not all are made equally, and these chemical composition changes could have important nutritional implications. Overall, it has been observed from the studies above that the inclusion of whey in dairy cattle diets to an extent could be a potential way to maintain or improve productivity. However high inclusion levels could decrease production performance.

## FEEDING STATEGY

As summarized in the studies above, we see that sugars can be included in the diet to maintain milk yield and fermentation parameters, while sometimes increasing DMI and NDF digestibility. However, it is important to note that in some literature when higher levels of inclusion where utilized there were adverse effects to fermentation and production. Hall (2002) noted that anecdotally adding sugars seemed to benefit most rations that had a low base sugar level and suggested that total sugar concentration in the lactating dairy cow diet should be around 5%. This would include the basal sugar already in the formulated diet and the sugar added as well. As previously mentioned, Broderick and Radloff (2004) further specified based on conclusions from the trial that diets should contain 5% total sugar, 2.4% of sugar from added sugar, and 2.6% of sugar present in the basal diet since overfeeding sugar appeared to reduce performance.

In the study by Brito et al (2014), corn meal or liquid molasses were fed in combination with either soybean–sunflower meal mix or flaxseed meal to mid-lactation Jersey cows. The inclusion rate of molasses was 12% of DM. The authors reported reduced yield of milk fat, ECM, and 4% FCM. They attributed the reduced animal performance to the 7.5% greater sugar inclusion than recommended level of 5.0%. In another experiment Ghendini et al (2017), ground corn was replaced with incremental levels of molasses in diets with flaxseed meal. The levels of inclusion were 0%, 4%, 8%, and 12% of DM to make four diets to be fed to 16 jersey cows in a replicated 4 × 4 Latin Square. The author reported decrease DMI in the cows fed increased amount of liquid molasses and decreased yield for milk, ECM, and 4% FCM, this was attributed to the excess amount of sugar included in their diets. The 5% inclusion threshold was surpassed at the 4% liquid molasses inclusion.

Overall, it is advantageous to have several feedstuffs available to feed cattle. It allows for more options when one feedstuff becomes less available or more expensive. Starch could be successfully replaced with byproducts to an extent because byproducts can maintain production and can be available when other feedstuffs are less abundant. Whey or whey permeate is a viable source for dairy producers who are near cheese plants as this would be an efficient way of utilizing whey byproduct. Therefore, apart from the benefits that feeding sugars in substitution for starch provide to animal production, their addition to the diet helps ensure that diets will meet animal energy requirements. It is also important to consider the inclusion of potential nitrogen sources in diets as with more readily available carbohydrates that are included in the diet available nitrogen sources such as urea should be considered for inclusion as well.

### Gaps in knowledge

When incorporating sugars into the diet there are still some topics that need to be addressed. One is when classifying byproducts to be added into the diet there is not a clear determination of composition in these byproducts. The study by Palmonari et al. (2020) was an experiment to help characterize differences in the chemical composition of beet vs. cane molasses, since these by products have been previous identified in the literature mostly by their DM, WSC, CP, and ash. The study emphasizes the importance of a more accurate description of molasses for it to be optimized when included in the diet because there can be difference in organic acids and mineral compositions which can drive differing results seen across experiments. Thus, sound reporting and product tags for composition may be important in better identifying performance difference.

Another interest is the incorporation of feeding sugars to calves in order to stimulate microbial proliferation and rumen development and epithelial growth through increased VFA production (Saegusa et al., 2017). Additionally, there is interest in incorporating sugars in the postpartum period of the transition period to aid cows in a state of negative energy balance (Caputo Olivera et al., 2019). These are both areas that merit further research to determine if there are benefits to the inclusion of sugars in the diet and to what extent they should be incorporated.

## CONCLUDING REMARKS

It has been observed that the replacement of starch with sugars is a viable option for producers due to positive effects on animal production and they also provide a feed option when commonly fed feedstuffs such as corn become less available or more expensive. Utilizing more byproducts can help reduce environmental impact and improve farm profitability. Sugars sources such as molasses (sucrose) and whey (lactose) have the potential to replace starch in dairy cow diets without adversely affecting the ruminal environment or milk yield and composition. It has been observed that the addition of sugars in the diet to a total of 5% of the DM could have positive effects in ruminal fermentation and milk production. Future steps for feeding sugars in the diet will further consider diet basal levels of sugar when incorporating additional sugar from byproducts in the diet.
